# Impacts of Natural Organic Matter Adhesion on Irreversible Membrane Fouling during Surface Water Treatment Using Ultrafiltration

**DOI:** 10.3390/membranes10090238

**Published:** 2020-09-17

**Authors:** Fangshu Qu, Zhimeng Yang, Shanshan Gao, Huarong Yu, Junguo He, Hongwei Rong, Jiayu Tian

**Affiliations:** 1 School of Civil Engineering, Guangzhou University, Guangzhou 510006, China; qufs@gzhu.edu.cn (F.Q.); 2111816241@e.gzhu.edu.cn (Z.Y.); huarongyu@gmail.com (H.Y.); junguohe@263.net (J.H.); rhwcn@vip.163.com (H.R.); 2 School of Civil and Transportation Engineering, Hebei University of Technology, Tianjin 300401, China; gaoshanshanhbu@163.com

**Keywords:** ultrafiltration, membrane fouling, natural organic matter, adhesion, background ions

## Abstract

To understand impacts of organic adhesion on membrane fouling, ultrafiltration (UF) membrane fouling by dissolved natural organic matter (NOM) was investigated in the presence of background cations (Na^+^ and Ca^2+^) at typical concentrations in surface water. Moreover, NOM adhesion on the UF membrane was investigated using atomic force microscopy (AFM) with colloidal probes and a quartz crystal microbalance with dissipation monitoring (QCM-D). The results indicated that the adhesion forces at the NOM-membrane interface increased in the presence of background cations, particularly Ca^2+^, and that the amount of adhered NOM increased due to reduced electrostatic repulsion. However, the membrane permeability was almost not affected by background cations in the pore blocking-dominated phase but was aggravated to some extent in the cake filtration-governed phase. More importantly, the irreversible NOM fouling was not correlated with the amount of adhered NOM. The assumption for membrane autopsies is doubtful that retained or adsorbed organic materials are necessarily a primary cause of membrane fouling, particularly the irreversible fouling.

## 1. Introduction

Because of outstanding advantages such as low-pressure running, small footprint, and high compatibility with other water purification technologies, ultrafiltration (UF) is increasingly applied in potable water treatment to meet stringent requirements for water quality. In particular, UF can totally retain particles, bacteria, and pathogens, therefore substantially improving water security [1]. However, deposition of natural organic matter (NOM), which is ubiquitous in surface water, usually causes severe membrane fouling in long-term operation, resulting in increased energy demand, and thus restricted engineering applications [2].

NOM fouling of UF membranes is very complicated due to the heterogeneous composition of NOM which is specific to water quality, type of membrane material, running conditions, and solution chemistry [3,4]. On the basis of experimental investigations throughout the world, colloidal and hydrophilic fractions of NOM are regarded to be the dominant foulants of the UF membranes, and therefore dissolved organic matter is involved to some extent via adhesion inside membrane pores [5,6]. In some cases, the amount of biopolymers, which are ubiquitous in natural water and depend on the biological activity, is used as an index to predict the severity of membrane fouling because surface water is commonly subject to microorganism contamination or algae blooms [7,8,9].

In addition to NOM, coexisting cations, particularly Na^+^ and Ca^2+^, in surface water play a significant role in the formation of NOM fouling. Essentially, the presence of cations can weaken electrostatic repulsion at interfaces via charge screening, double layer compression, bridging effects, or complexation according to the classical Derjaguin–Landau–Verwey–Overbeek (DLVO) theory [4]. On the one hand, the electrostatic repulsion at the foulant–membrane interface prevents the negatively charged organics from adhering to the negatively charged membrane surface. Membrane fouling is, thus, aggravated in the presence of cations during filtration of either surface water or secondary effluent [10,11,12]. Furthermore, in a filtration test of surface water with different types of cations (Ca^2+^ and Mg^2+^) at different concentrations, a linear relationship was observed between the irreversible fouling rate and concentration of cations [13,14]. On the other hand, electrostatic repulsion at the foulant–foulant interface is also reduced in the presence of cations. Foulants aggregate to an extent specific to the type and amounts of cations. The aggregates deposit on the membrane during filtration and form a cake layer with more void space, resulting in increased membrane permeability [15]. In a study on alginate recovery using UF, the filterability of the feed was demonstrated to exponentially increase with increasing Ca^2+^ concentration in a range of 0–10 mM [16]. Hence, the observed membrane fouling in the presence of cations is the tradeoff between aggravated fouling due to enhanced foulant–membrane interactions and alleviated fouling because of the strengthened foulant–foulant interaction, which explains the inconsistent results reported in the literature.

Recently, further investigations on the impact of cations on UF membrane fouling by NOM have been performed in a broad concentration range for cations such as Na^+^, Ca^2+^, and Mg^2+^. Progress has been made in understanding the impact of cations on the migration of foulants and fouling behaviors during filtration, particularly for polysaccharides. Biphasic fouling behavior has been demonstrated with increasing concentrations of cations. At low concentrations, Ca^2+^ ions preferentially interact strongly with guluronate functional groups, forming a highly interconnected gel assemblage with egg-box structure; despite the highly porous structure, the fouling layer shows very high resistance to water flow due to the accelerated crosslinking of the alginate gel layers [17]. With an increasing concentration of cations, intramolecular binding outperforms intermolecular binding within the alginate, resulting in the formation of alginate-Ca clusters, which deposit on the membrane surface, forming a cake layer characterized by a lower porosity and, most importantly, a lower specific resistance to the passage of water [18,19]. Moreover, it has been proposed that there exists a critical calcium concentration, of 3 mM, below which increasing calcium concentration leads to more severe fouling and above which fouling is increasingly mitigated [20]. Miao et al. [21] reported that the biphasic impact of cations on alginate fouling also applied to fouling by humic acids, which was aggravated at an ionic strength below 2.5 mM and alleviated as the ionic strength exceeded 5 mM. However, these works were typically performed with model foulants such as alginate, proteins, or humic acids rather than organics in natural surface water. Jiang et al. [22] compared the colloid properties of sodium alginate and polysaccharides in extracellular polymeric substances (EPS) with regard to membrane fouling and found that the substitution of polysaccharides in EPS with alginate for membrane fouling studies was not convincing. Hence, a fundamental investigation on the impact of background cations on the fouling behavior of NOM is still warranted to better understand membrane fouling during surface water treatment using UF, particularly at typical cation concentrations in surface water. Moreover, of particular interest is whether or not NOM adhesion, which substantially varies with the background cations, is a significant cause of irreversible fouling. This is regarded to be the fundamental logic for foulant identification by membrane autopsies.

In this work, NOM in surface water was extracted by reverse osmosis concentration and dialysis desalination was used to remove background ions. Filtration tests were performed with the reconstituted NOM solutions; the removal of NOM and fouling extent during filtration were assessed. Moreover, the membrane-NOM interaction and the adhesion of NOM onto the membrane were investigated using AFM coupled with a probe and a quartz crystal microbalance with dissipation monitoring (QCM-D), respectively, to obtain more insight into the fouling behavior of NOM at typical concentrations of Na^+^ and Ca^2+^ in surface water.

## 2. Materials and Methods

### 2.1. Raw Water and Natural Organic Matter (NOM) Extraction

Natural water was sampled from the Songhuajiang River in Harbin, China. The raw water was immediately filtered through a 0.45 μm mixed cellulose filter (Taoyuan Co. Ltd., Haining, China) to remove solid particles and NOM colloids with large size. The raw water quality is summarized in Table S1 in Supplementary Materials. Because of severe organic pollution, high amounts of NOM occurred in the water with varying concentrations of dissolved organic carbon (DOC) ranging from 5.41–7.55 mg L^−1^ and values of UV absorbance at 254 nm (UV_254_) from 0.172 to 0.218 cm^−1^. Other water quality indexes for the raw water are found in Table S1 in Supplementary Materials. The rejection rate is defined as percentage of foulants that are retained by the membrane over the whole filtration. NOM extraction was performed in 3 steps. First, the raw water was filtered by the 0.45 μm mixed cellulose filter to remove suspended particles. Second, the filtrate was collected and concentrated by 20 times using reverse osmosis (GCM-S-02, Guochu technology, Xiamen, China). The recovery of organics was approxunatekt 80%. Third, the concentrate was desalinated to remove the ions using dialysis membrane bags (MD34, Union Carbide, Connecticut, USA) [23]. The MWCO of the dialysis membrane was 3000 Da. The desalination was continuously performed with dialysis bags replaced every 6 h, until the conductivity was less than 200 μS/cm. Finally, the water samples were stored at 4 °C and used within 72 h.

### 2.2. Membranes and Experimental Setup

Flat (PES) UF membranes (OM100076) were purchased from Pall Co. (Millipore, MI, USA). The membranes were made of polyethersulfone and had a nominal molecular weight cutoff of 100 kDa. A bench-scale filtration system, which was introduced elsewhere [24], was used in this work. As shown in Figure S1, the filtration system comprised a nitrogen gas cylinder, two UF cells (Amicon 8400, Millipore, MI, USA), two electronic balances, a computer, a pressure gauge, and three valves. The membrane was placed in the bottom of the cell with its skin (glossy) side upward to the feedwater. The electronic balance automatically logged the weight of the filtrate every 5 s. The filtration was performed in a dead-end mode, which was widely used in potable water treatment due to fewer foulants in surface water as compared with wastewater.

The feedwater was prepared by diluting the concentrated natural water to a DOC concentration of 6 mg/L. The feedwater and permeate were sampled to investigate the rejection of NOM by UF. In addition, the feedwater was spiked with NaCl or CaCl_2_ to understand the effects of background ions in surface water on membrane fouling. The concentrations of Na^+^ and Ca^2+^ were 15 and 18 mg/L, respectively, which were identical to the concentrations in the surface water. The membrane fouling was assessed by three types of resistance, i.e., intrinsic membrane resistance, reversible resistance, and irreversible resistance, which were related to the pore characteristics, cake layer formation due to foulant deposition, and pore blocking due to foulant adhesion inside membrane pores, respectively. The fouling resistance was evaluated via the resistance–in-series model.

Briefly, filtration of 300 mL milli-Q water under 60 kPa was performed in each filtration test to determine the initial membrane flux. Subsequently, 400 mL water was fed into the UF cell and 350 mL was filtered at a constant pressure of 60 kPa. The flux was monitored to investigate the membrane fouling. When filtration was terminated, the fouled membranes were turned over and flushed with Milli-Q water in the cell for 2 min under 60 kPa. Finally, filtration of milli-Q water was performed again with the backwashed membranes to determine fouling reversibility. Each filtration test was performed in triplicate. The intrinsic membrane resistance, reversible resistance, and irreversible resistance during filtration were obtained via Equations (1)–(3) [25].

(1) $$R_{i}=\frac{\Delta P}{{\mu J}_{0}}$$

(2) $$R_{ir}=\frac{\Delta P}{{\mu J}_{w}}-\frac{\Delta P}{{\mu J}_{0}}$$

(3) $$R_{re}=\frac{\Delta P}{{\mu J}_{w}}-\frac{\Delta P}{{\mu J}_{e}}$$

where Δ*P* is the operating pressure (Pa), *μ* is the dynamic viscosity (Pa s), *J*_0_ is the initial flux for the UF membrane, *J_e_* is the flux at the end of filtration, and *J_w_* is the clean water flux of the backwashed membrane.

### 2.3. Analytical Methods

Membrane-based size fractionation and nonionic resin isolation were used to characterize the NOM in the surface water. The details for the methods were induced elsewhere [15]. Briefly, five types of regenerated cellulose membranes (Millipore, MI, USA) with molecular weight cut-offs (MWCOs) of 100, 30, 5, 3, and 1 kDa were used to separate NOM into fractions of >100, 30–100, 5–30, 1–5, and <1 kDa. Fractionation was performed under a pressure of 100 kPa with a stirrer running at 250 rpm to reduce concentration polarization and cake layer formation. For each fraction, a 100 mL water sample was fed into the stirred cell and 50 mL permeate was produced; the initial 10 mL was discarded. The XAD resins were sequentially washed with 0.1 N NaOH, 0.1 N HCl, methanol, and Milli-Q water until no additional organic compounds were detected in the rinsing effluent [26]. The water samples were acidified to pH = 2 and successively pumped into the DAX-8 and XAD-4 resin columns. The hydrophobic and transphilic fractions were adsorbed onto the DAX-8 resin and XAD-4 resin, respectively, whereas the hydrophilic fraction passed through both resins [27]. The flow rate was set at 15 wet resin volumes per hour. Fractionation tests were performed in triplicate with errors less than 5%.

A fluorescence spectrophotometer (F7000, Hitachi, Tokyo, Japan) was used to obtain three-dimensional (3D) fluorescent excitation-emission matrix (EEM) fluorescence spectra of the water samples. The FEEM measurements were performed at excitation wavelengths of 220 to 450 nm in 5 nm increments and at emission wavelengths of 250 to 550 nm with 1 nm increments. A photomultiplier tube (PMT) voltage of 700 V and a scanning speed of 2400 nm min^−1^ were adopted. Measurements were taken at room temperature (21 ± 1 °C). The absorbance at 254 nm of each sample was measured using a UV/Vis spectrophotometer (T6, Puxi, Beijing, China) beforehand. Samples with UV_254_ values exceeding 0.05 cm^−1^ were diluted with Milli-Q water to minimize the inner filter effect, and the dilution factor of each sample was recorded [28]. The fluorescence spectrum of Milli-Q water was subtracted from all spectra to eliminate water Raman scattering. Moreover, the fluorescence data were further interpreted by the parallel factor (PARAFAC) analysis, of which the details are introduced in Supplementary Materials [29].

An atomic force microscope (AFM, Bruker, Santa Barbara, CA, USA) in conjunction with a SiN cantilever (Bruker NP-O10, Bruker, Santa Barbara, CA, USA, spring constant 0.06 N m^−1^) was used to access interfacial adhesion force between NOM and the UF membrane according to Miao et al. [30]. The free end of a tipless cantilever probe was coated with a small amount of epikote. Then, the cantilever was immersed in a concentrated NOM solution, for 24 h, to prepare the NOM coated probe. Finally, to strengthen the connection between the NOM microsphere and the cantilever, the NOM colloidal probes were stored at 4 °C, for at least 1 week, prior to use. Diluted NOM solutions with and without background ions were used as the testing solutions. The AFM interaction force measurements were performed in a fluid cell using a closed inlet/outlet loop under contact mode. A clean membrane was installed at the bottom of the fluid cell which was flushed and filled with the testing solution. According to the Derjaguin approximation, the intensive property (F/R) between a flat plate and a sphere is twice the magnitude of the corresponding force exerted between two identical spheres [31]. For each membrane sample, at least 16 locations were randomly chosen for force measurements, and more than 10 force curves were obtained at each location.

Moreover, the adhesion of NOM on the UF membrane was evaluated by a quartz crystal microbalance with dissipation monitoring (QCM-D) (E1, Qsence, Götenborg, Sweden) combined with a PES-coated sensor crystal. Specifically, a small amount of epoxy resin was coated on a clean sensor crystal via a spin coater (EZ4, Schwan Technology, Marshall, MN, USA). The coating was performed at a speed of 3000 rpm for 60 s. A clean membrane was cut and pasted onto the coated sensor crystal. Then, the membrane-coated sensor crystal was rinsed with milli-Q water and dried under pure nitrogen gas for approximately 2 min. For adhesion tests, the sensor crystal was installed in the QCM-D, and milli-Q water flowed through the measurement chamber until a stable baseline was achieved. The temperature was maintained at 23 °C and the flow rate was set at 0.1 mL/min with a peristaltic pump (BW100, Longer, Baoding, China). After that, the testing solution was shifted to the reconstituted NOM solutions. The measurement consisted of two parts, i.e., an adsorption process and desorption process. The NOM solutions and milli-Q water were used as the testing solutions in the adsorption process and a desorption process, respectively. During measurements, both the frequency and dissipation were monitored and recorded to assess the amount of adsorbed foulants and the structure of the adsorption layer, respectively. According to the Sauerbrey relationship (Equation (4)), the crystal sensor frequency is negatively correlated with the adsorbed mass; the dissipation reflects the energy dissipation of adsorbed foulants during deposition and provides insight into the structure of deposit layers [32] as:

(4) $$\Delta m=-\frac{C}{n}\Delta f$$

where Δ*m* is the adsorbed mass, Δ*f* is the frequency, *n* is the overtone number, and *C* is the crystal constant.

A total organic carbon (TOC) analyzer (multi N/C 2100S, Jena, Jena, Germany) was used to determine the concentrations of DOC in the water samples. Calibration was performed with potassium hydrogen phthalate and a linear correlation (0–20 mg L^−1^) was obtained with an *R*^2^ value of 0.999. An ultraviolet (UV) spectrometer (T6, Puxi, Beijing China) was used to measure the UV_254_ values of the water samples. The water samples were prefiltered by a 0.45 μm filter to remove suspended particles prior to measurements. The filters were cleaned in boiled water for 20 min to minimize leached organics, and the first 10 mL filtrate was discarded. For SEM observation, membrane samples were coated with an approximately 20 Å Au/Pd layer using a precision etching coating system (Model 682, Gatan, Pleasanton, CA, USA), and then observed under SEM (Quanta 200FEG, Eindhoven, Netherlands). The membrane samples were fractured in liquid nitrogen and naturally dried under ambient conditions.

## 3. Results and Discussion

### 3.1. Characteristics of NOM in Surface Water

**Figure 1:**
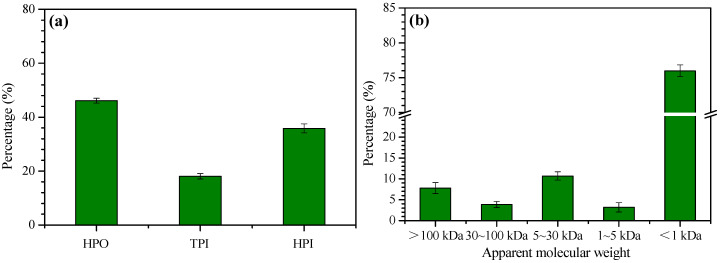
Hydrophobicity (**a**) and molecular weight distribution; (**b**) of natural organic matter (NOM) in raw water. Error bar indicates standard error of triplicate measurements.

The characteristics of NOM have great implications for the membrane fouling during filtration. Figure 1a shows the fractionation results for the dissolved NOM in the Songhuajiang River by DAX-8 and XAD-4 resins. It can be observed that the hydrophobic, transphilic, and hydrophilic fractions account for 46%, 18%, and 36%, respectively, of the total organics in the dissolved NOM in terms of DOC. Figure 1b shows the size fractionation results for NOM in the surface water. The majority of the NOM (76%) is less than 1 kDa in molecular weight. The fractions of >100 kDa, 30–100 kDa, 5–30 kDa, and 1–5 kDa only account for 8%, 4%, 11%, and 3%, respectively, of total NOM. In brief, the dissolved NOM was characterized by more hydrophobic and low-molecular weight fractions.

### 3.2. Effects of Background Cations on Interfacial Interactions between the NOM and Membrane Surface

**Figure 2:**
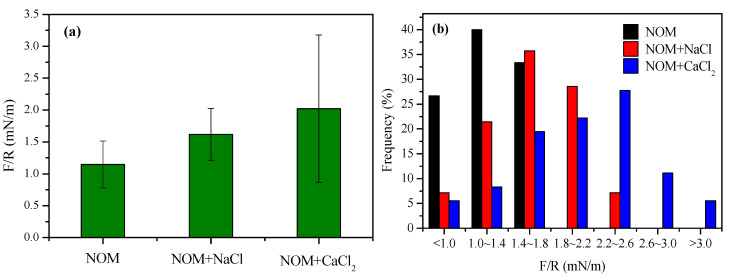
(**a**) Interfacial interactions between the reconstituted NOM and membrane surface in the absence or presence of cations: Interacting adhesion force; (**b**) Distribution of adhesion force. Error bars indicate the standard error of 160 measurements.

Figure 2 shows the average values and distribution of interacting forces between NOM and the PES membrane. The adhesion forces were normalized by the radius of the colloidal probe used. The specific adhesion force between the reconstituted NOM and the membrane was determined to be 1.15 mN m^−1^. In the presence of Na^+^ and Ca^2+^, the specific adhesion forces increased to 1.62 and 2.02 mN m^−1^, respectively, implying that the background cations, particularly divalent Ca^2+^, in surface water can enhance the potential for NOM adhesion onto the membrane surface. This is consistent with the reported results in the literature. Miao et al. [33] reported that the adhesion forces between humic acids and a PVDF membrane substantially increased with increasing concentrations of background cations such as Na, Ca^2+^ and Mg^2+^. Adhesion forces represent electrostatic interactions, acid−base interactions, hydrogen bonding, and van der Waals interactions. The presence of cations reduced electrostatic repulsion between foulants and the membrane surface due to screening effects and double layer compressions and resulted in increased adhesion forces. In another study on NF fouling by Suwannee River NOM, the adhesion force was remarkably correlated with fouling rate in initial filtration phases [34].

### 3.3. Effects of Background Cations on NOM Adhesion onto the Ultrafiltration (UF) Membrane Surface

**Figure 3:**
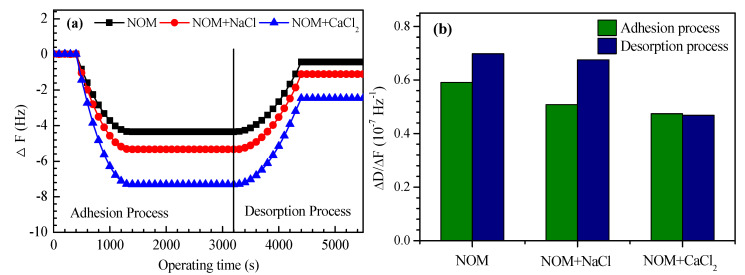
Normalized change in Δ*F* (**a**) with time and absolute values of Δ*F/*Δ*D*; (**b**) for NOM adhesion on a flat (PES) membrane in the absence or presence of background cations. The dissolved organic carbon (DOC) and pH of the reconstituted NOM were 10 mg/L and 7.0 ± 0.2, respectively. The adhesion test was performed at an ambient temperature of 21 °C, and a flow rate of 0.1 mL min^−1^.

The adhesion of the reconstituted NOM on the PES membrane was assessed by QCM-D; the changes in the oscillating frequency (Δ*F*) of the quartz crystal and the energy dissipation (Δ*D*) describe the amount of adsorbed organics and the structural properties of the fouling layer, respectively [35]. As shown in Figure 3a, Δ*F* substantially increased as the reconstituted NOM solution was fed and gradually reached a plateau with a Δ*F* value of 4.35 Hz, implying that the adhesion of NOM on the PES membrane was saturated. In the presence of Na^+^ and Ca^2+^, the Δ*F* values increased to 5.34 and 7.30 Hz, respectively. This is consistent with the adhesion force results, in which the maximum adhesion force was observed in the presence of Ca^2+^. It can be inferred that background cations, particularly Ca^2+^, substantially enhanced the adhesion of NOM on the PES membrane due to reduced electrostatic repulsion. Wang et al. [36] investigated membrane fouling by humic acids using QCM-D and found that the bridging effects of Ca^2+^ and Mg^2+^ promoted the assembly and deposition of humic acids on the PES surface, which resulted in accelerated foulant accumulation. When the test solution shifted to milli-Q water, the Δ*F* values in all three cases started to decrease, indicating the desorption of organics from the membrane surface. However, the increased Δ*F* values could not be totally restored. The residual Δ*F* values were 0.43 Hz in the absence of background cations, implying that some NOM adhesion was irreversible. In the presence of Na^+^ and Ca^2+^, the residual Δ*F* values were 1.11 and 2.46 Hz, respectively. The irreversible adhesion of NOM was aggravated in the presence of background ions.

Figure 3b presents the variations in the absolute Δ*D/*Δ*F* values, which are associated with the structural properties of adhesion layers, in the adhesion and desorption processes. A lower absolute value of Δ*D/*Δ*F* implies a dense and compact layer, whereas a higher value describes a loose and dissipative layer [37]. The absolute values of Δ*D/*Δ*F* were 0.591 and 0.698 in the adhesion and desorption processes, respectively, implying that the residual layer was much looser than the adhesion layer. In the presence of Na^+^ and Ca^2+^, the absolute values of Δ*D/*Δ*F* decreased to 0.508 and 0.474, respectively, in the adhesion process. This result indicates that the adhesion layer on the membrane surface becomes denser and more compact in the presence of background cations, which is attributed to tighter NOM binding and aggregation. Ferrando et al. [38] investigated fouling in reverse osmosis by extracellular polymeric substances and found that the presence of Ca^2+^ led to an increase in both the adsorption and rigidity of the adsorbed layer.

### 3.4. Effects of Background Cations on the Rejection of NOM by UF

**Figure 4:**
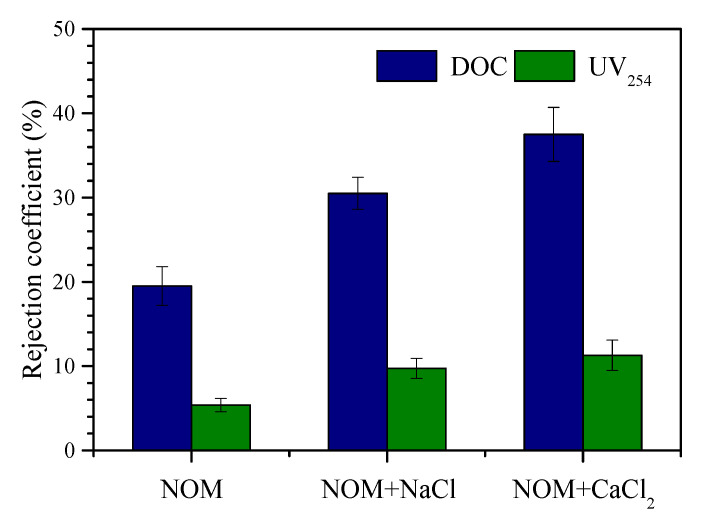
Performance of organic rejection during treatment of reconstituted NOM solution using ultrafiltration (UF). The DOC concentration and UV_254_ of the feed water were 5.72 mg L^−1^ and 0.187 cm^−1^, respectively. Error bars indicate the standard error of triplicate measurements.

Figure 4 shows the rejection of NOM with respect to DOC and UV_254_ during surface water treatment using UF. The rejection coefficients for DOC and UV_254_ were 19% and 5%, respectively, during filtration of the reconstituted NOM solution. The DOC rejection was higher than that expected based on the molecular weight distribution of NOM (Figure 1b), because size exclusion as well as membrane absorption and cake layer retention were involved in the removal of organics during filtration [24]. In the presence of Na^+^, the rejection performance was improved, with rejection coefficients increasing to 31% and 10% for DOC and UV_254_, respectively. The DOC rejection by UF was further increased to 38% in the presence of Ca^2+^. The results imply that the occurrence of cations contributes to improved NOM rejection during surface water treatment using UF. The NOM rejection in the presence of background cations is positively correlated with the adhesion forces and QCM-D results. On the one hand, cations can enhance the aggregation of organics, particularly negatively charged fractions, by reducing intermolecular electrostatic repulsion and thus improve NOM rejection based on size exclusion [39]. On the other hand, the electrostatic repulsion between the membrane and organics is also reduced in the presence of cations, particularly Ca^2+^, resulting in reduced organic penetration [40].

**Figure 5:**
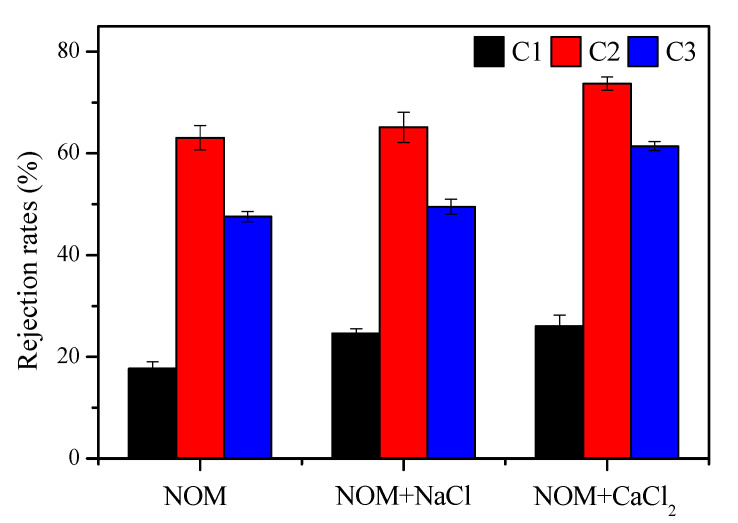
Performance of fluorescent component rejection during reconstituted NOM solution treatment using UF. Error bars indicate the standard error of triplicate measurements.

To gain more insight into NOM removal, FEEM was used to characterize fluorescent components in the feed and UF permeate. The FEEM spectra for the feedwater and permeate samples are shown in Figure S2. Moreover, the FEEM data were further analyzed by parallel factor modeling, which is introduced in Supplementary Materials, The wavelength pairs for the identified components in the FEEM spectra of NOM (Figure S3) are shown in Table S2. Three components, denoted as C1 (Ex/Em: 230(300) nm/412 nm), C2 (Ex/Em: 230(280) nm/330 nm), and C3 (Ex/Em: 260(350) nm/465 nm), were associated with microbial humic-like compounds, protein-like compounds and terrestrial humic-like substances, respectively. The fluorescent intensities for these components were used to calculate the rejection rates, and the results are shown in Figure 5. For reconstituted NOM without cations, the rejection rates for C1, C2, and C3 were 18%, 63%, and 48%, respectively, implying the more favorable removal of protein-like and terrestrial humic-like substances. Parts of protein-like compounds, which are biopolymers excreted by microorganisms, are high in molecular weight, and thus favorably retained by UF [41]. Humic-like fractions are typically strong in hydrophobicity and can be absorbed on the membrane surface [42]. Hence, the rejection performance for fluorescent components was generally better than the DOC rejection performance. In the presence of Na^+^ and Ca^2+^, the rejection of C1, C2, and C3 was generally improved to some degree, and the maximum removal was observed for C2 (protein-like compounds). This is attributed to reduced electrostatic repulsion and increased organic adhesion on the membrane surface in the presence of cations, which is generally consistent with the QCM-D results.

### 3.5. Effects of Background Cations on Membrane Fouling Caused by NOM

**Figure 6:**
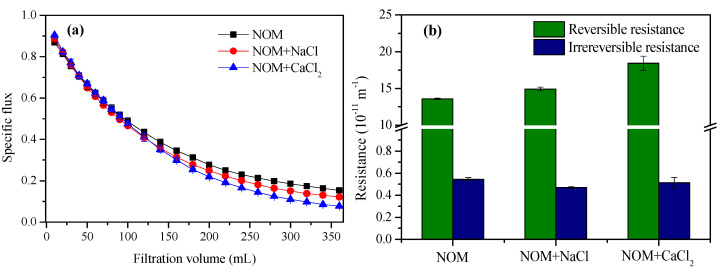
Specific fluxes (**a**) and fouling resistances; (**b**) during filtration of the reconstituted NOM solutions. The DOC concentration and pH value of the feedwater were 6.0 mg L^−1^ and 7.0 ± 0.2, respectively. The error bars indicate the standard deviation (n = 3).

Figure 6 shows the flux profiles and resistance during filtration of the reconstituted NOM solutions. In the absence of cations, the membrane was severely fouled, with the flux decreasing by 52% after filtering 100 mL NOM solution, and the flux decline gradually slowed, with a specific flux of 0.15 at the end of filtration. As shown in Figure S4 in Supplementary Materials, the fouling process was successively dominated by pore blocking and cake filtration in the initial filtration phase and the later filtration phase, respectively. In the presence of Na^+^ and Ca^2+^, the flux decline in the initial phase was almost identical to that with NOM alone, regardless of the increased NOM adhesion (Figure 4). The probable interpretation is that the monolayer adhesion of organics inside pores or on the membrane surface, which occurs in a very short period of time, plays a minor role in fouling development. As the filtration proceeded into the cake filtration-dominated phase, the flux decline was slightly aggravated in the presence of background cations. The specific fluxes at the end of the filtration of the reconstituted NOM solutions were 0.12 and 0.08 in the presence of Na^+^ and Ca^2+^, respectively. This is consistent with the increased NOM adhesion and more compact fouling layer (Figure 3) in the presence of background cations. According to the DLVO theory, electrostatic repulsion between foulants can be reduced in the presence of cations due to screening effects and double layer compression, resulting in increased adhesion forces (Figure 2), and thus tighter binding of foulants. In another similar work, the pseudostable flux of a UF membrane fouled by proteins decreased in the presence of Na^+^ within a low ionic strength range (<1 mM) [43]. Overall, the flux decline was slightly aggravated by the presence of background cations at typical concentrations in surface water, and the membrane permeability was dependent on both the amount of NOM deposited and the structure of the fouling layer.

As shown in Figure 6b, the reversible and irreversible resistance was 13.56 and 0.54 × 10^11^ m^−1^, respectively, during filtration of the reconstituted NOM solution. The reversible resistance increased to 14.91 × 10^11^ m^−1^ and 18.42 × 10^11^ m^−1^ in the presence of Na^+^ and Ca^2+^, respectively, which is consistent with the variations in membrane flux. It is known that Ca^2+^ can preferentially reduce foulant–foulant rather than membrane–foulant electrostatic repulsion, resulting in enhanced aggregation of foulants, and thus formation of a more porous cake layer [44]. However, reversible fouling was aggravated rather than retarded in the presence of the background cations in this work, especially with Ca^2+^. Moreover, as shown in Figure S4, the pore blocking-dominated filtration phase was extended in the presence of the cations, and the shift of the dominant mechanism to cake filtration was delayed. The contradiction probably originated from the low concentrations of the background cations in surface water. It has been demonstrated that, for alginate solution, the specific filtration resistance presented a unimodal pattern variation with increasing Ca^2+^ concentration, because the initial increase in Ca^2+^ concentration facilitates intermolecular rather than intramolecular binding, resulting in a more homogeneous gel layer and higher resistance, whereas excess Ca^2+^ induced the formation of alginate calcium clusters and less filtration resistance due to the macrovoid pores in the interclusters [19,20]. The Ca^2+^ concentration in the surface water was not sufficient to cause intramolecular binding for organics, therefore, NOM fouling was aggravated in the presence of Ca^2+^ in this work. In another similar work, reversible membrane fouling by humic acids was also aggravated in the presence of divalent cations (Ca^2+^ and Mg^2+^) at concentrations lower than 10 mg Ca^2+^ mg^−1^ DOC [21]. Regarding irreversible fouling, the resistance was slightly reduced to 0.47 and 0.52 × 10^11^ m^−1^ in the presence of Na^+^ and Ca^2+^, respectively, which was inconsistent with the increased adhesion force (Figure 2) and increased amount of residual NOM on the crystal sensors (Figure 3). This result raises doubt regarding the presumption that retained or adsorbed organic materials are necessarily a primary cause of fouling. Kim and Dempsey [45] demonstrated an inverse relationship between absorbance in the infrared regions and resistance to filtration in an investigation on UF membrane fouling by dissolved organic matter in secondary effluent. The probable interpretation was that the compact fouling layer that formed in the presence of background cations acted as a prefilter, resulting in more organic retention in the fouling layer rather than direct deposition on the membrane surface [24]. Hence, the fundamental logic of foulant identification by membrane autopsies could be questioned. NOM deposition may not necessarily be a significant cause of irreversible fouling during surface water treatment using UF, and the effects of background cations and the properties of the fouling layer could play a role. It should be noted that limited types of NOM and membrane, and more investigations are still warranted with more water types and membranes.

### 3.6. Morphological Characterization of Fouled Membranes by NOM

**Figure 7:**
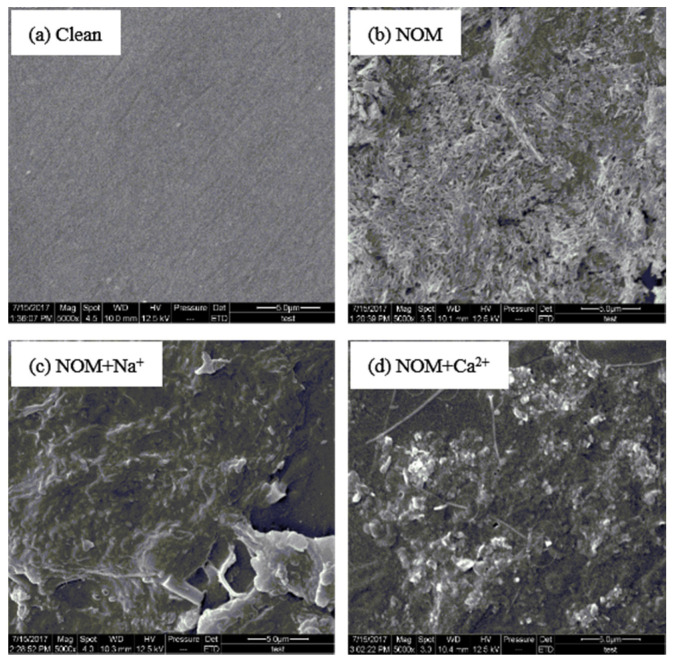
SEM images of fouled membranes after filtering reconstituted NOM solutions. (**a**) Control; (**b**) NOM; (**c**) NOM + NaCl; (**d**) NOM + CaCl_2_.

Figure 7 presents SEM images of new and fouled membranes. The new membrane was clean, and many tiny pores could be visually discerned. After filtering the reconstituted NOM solution, the membrane was covered by a thick fouling layer (Figure 7b), in which microscopic voids formed by overlapping organics could be discerned. In the presence of Na^+^ and Ca^2+^, thick and dense fouling layers were observed on the membrane surface, which was consistent with the increased reversible fouling resistance in the presence of background cations. This result further verified the unfavorable impact of background cations at low concentrations on membrane permeability. Moreover, the fouled membranes were characterized by FTIR, and the results are shown in Figure S5 in Supplementary Materials. In contrast, the characteristic absorbance peaks for new and fouled membranes were almost identical within the wavenumber range of 0–1740 cm^−1^. Beyond this range, typical peaks at approximately 3300 cm^−1^, which refer to the bending vibration of adsorbed molecular water and the stretching vibration of hydroxyl groups [46], were observed in the FTIR spectra of the fouled membrane. Overall, the presence of background cations at typical concentrations in surface water was generally favorable to NOM deposition during surface water treatment using UF.

## 4. Conclusions

In this work, membrane fouling was systematically assessed in the presence and absence of background cations at typical concentrations in surface water. The NOM-membrane adhesion forces were increased in the presence of Na^+^ and Ca^2+^ at typical concentrations in surface water, and the amount of adhered NOM increased due to reduced electrostatic repulsion, resulting in improved NOM retention. The membrane permeability was minimally affected by background cations in the pore blocking-dominated phase but aggravated to some extent in the cake filtration-governed phase. The presence of Na and Ca^2+^ increased the adhesion of NOM on the membrane, but irreversible NOM fouling was not correlated with the amount of adhered NOM.

## Supplementary Materials

The following are available online at https://www.mdpi.com/2077-0375/10/9/238/s1, Table S1: Quality of raw water taken from Songhuajiang river, Table S2: Wavelength pairs for three types of components identified in the FEEM spectra of NOM, Figure S1: A schematic diagram of the bench-scale UF system, Figure S2: Fluorescent characteristics of NOM in the raw water, Figure S3: Fluorescent components identified from NOM, Figure S4: *d*^2^*t*/*dV*^2^ vs. *dt/dV* curves for the filtration of reconstituted NOM solutions, Figure S5: FTIR spectra for fouled membranes after filtering reconstituted NOM solutions.
