# Supplementary material for: Impacts of Natural Organic Matter Adhesion on Irreversible Membrane Fouling during Surface Water Treatment Using Ultrafiltration
Source: Membranes (Basel). 2020 Sep 17;10(9):238. doi: 10.3390/membranes10090238 (PMC7557390; doi:10.3390/membranes10090238)
Supplement: Supplementary file 1 [file membranes-10-00238-s001.pdf]

Manuscript for possible publication in Membranes

Date: Aug. 21st 2020

Impacts of natural organic matter adhesion on irreversible membrane fouling during surface water treatment using ultrafiltration

Fangshu Qu[a], Zhimeng Yang[a], Shanshang Gao[b,*], Huarong Yu[a], Junguo He[a], Hongwei Rong[a], Jiayu Tian[b,*]

Supplementary Information

[a]School of Civil and Transportation Engineering, Hebei University of Technology, Tianjin 300401, China

[b]School of Civil Engineering, Guangzhou University, Guangzhou, 510006, P.R. China

*Corresponding author.

Tel.: +86 13928755563.

E-mail address: gaoshanshanhbu@163.com (Shanshan Gao);

tjy800112@163.com (Jiayu Tian);

1. Raw water quality

Table S1 Quality of raw water taken from Songhuajiang river

Indexes	Ranges
Temperature (ºC)	0-28
pH	7.53-7.81
Turbidity (NTU)	11.8-35.4
DOC (mg/L)	5.41-7.55
UV_{254} (cm^{-1})	0.172-0.218
Total count of bacteria (CFU)	< 3000
Total count of coliforms (MPN)	< 1200
K^+ (mg/L)	3.42
Ca^{2+} (mg/L)	20.83
Na^+ (mg/L)	15.12
Cl^- (mg/L)	8.89
SO_4^{2-} (mg/L)	17.28
NO_3^- (mg/L)	4.60

2. Experimental ultrafiltration (UF) system

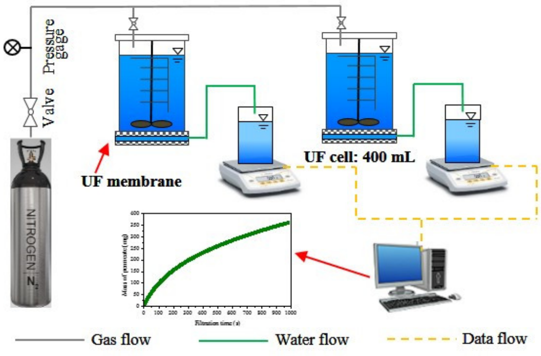

Fig. 1 A schematic diagram of the bench-scale UF system

3. **Fluorescent excitation-emission matrix spectrum (FEEM) of the reconstituted NOM solution and permeate samples**

FEEM was used to characterize fluorescent components in the feed and UF permeate. The FEEM spectra for the feedwater and permeate samples are shown in Fig. S2.

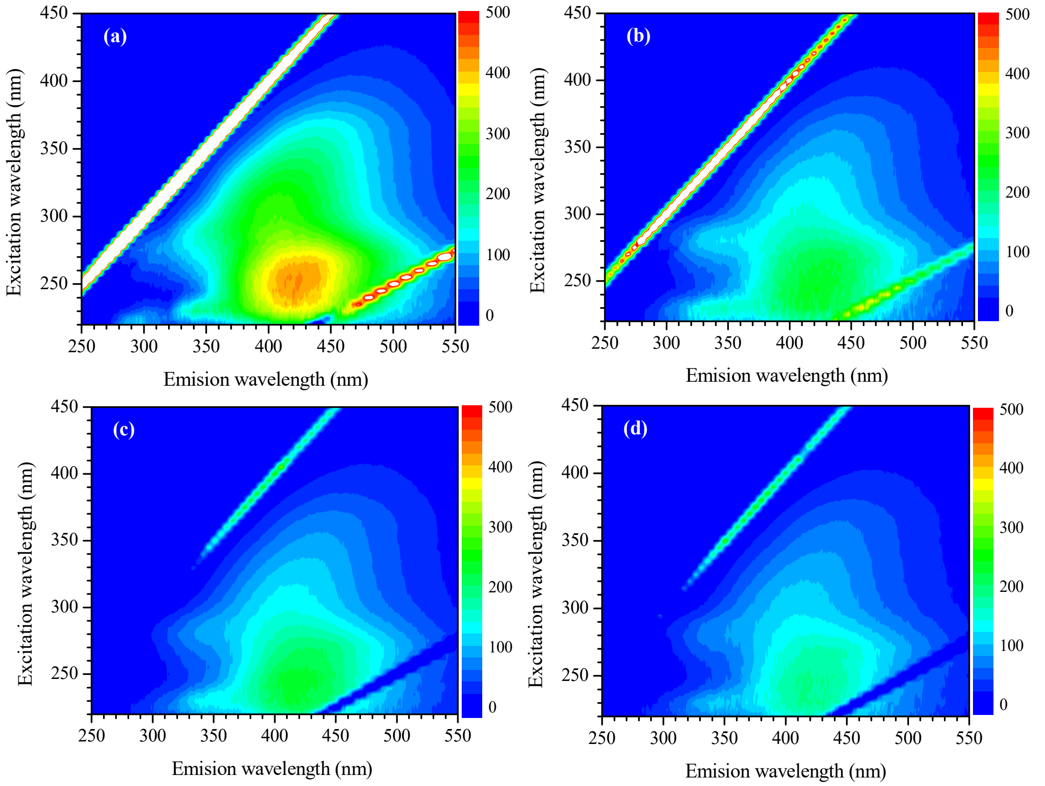

Fig. S2 Fluorescent characteristics of NOM in the raw water: (a) reconstituted NOM solution, (b) UF permeate in absence of the cations; (c) UF permeate in presence of Na^{+} and (c) UF permeate in presence of Ca^{2+}

4. Analyzing FEEM data of NOM via the Para-factor modeling

PARAFAC modeling procedures conducted were similar to that described by Stedmon and Bro [1]. Briefly, a dataset of 44 EEM fluorescence data were modeled using the DOMFluor Toolbox in Matlab® according to the recommended procedures [1]. With the PARAFAC analysis, spectrally overlapping EEM data can be mathematically separated into chemically independent fluorescence components. A series of PARAFAC models consisting of between 3 and 7 components were generated. The number of fluorescence components was identified by a validation method including residual analysis, split half analysis and random initialization analysis. Three components were identified in this work as shown in Fig. S3 and the wavelength pairs for the identified components are summarized in Table S1. Component 2 is in the region of protein-like fluorophores, whereas Component 1 and Component 3 are indicated to be humic-like fluorophores [2]. The maximum fluorescence intensity (F_{max}) of each component in each sample generated from PARAFAC model has been used to estimate the relative concentration of the corresponding component in a sample, and excitation and emission loadings indicated their characteristics excitation and emission spectra [2-4].

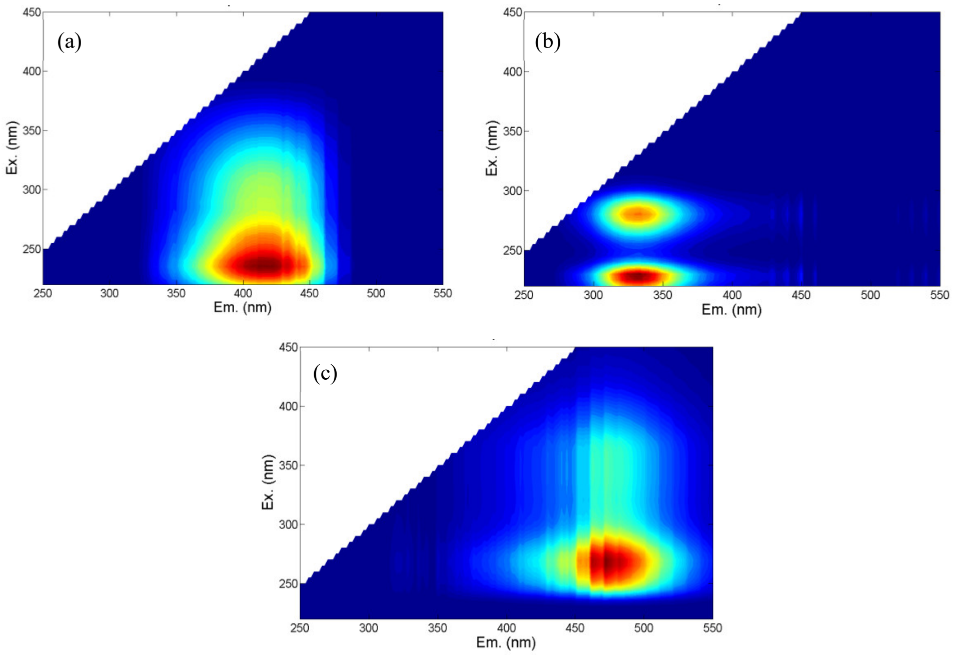

Fig. S3 Fluorescent components identified from NOM: (a) component 1, (b) component 2, and (c) component 3

Table S1 Wavelength pairs for three types of components identified in the FEEM spectra of NOM

Components	C1	C2	C3
Wavelength pairs	Ex: 230(300) nm<br>Em: 412 nm	Ex: 230(280) nm<br>Em: 330 nm	Ex: 260(350) nm<br>Em: 465 nm
Description	Microbial humic like	Protein-like	Terrestrial humic-like

5. Mechanisms for NOM fouling in the absence or presence of Na^+ and Ca^{2+}

To understand the mechanisms for EfOM fouling, the filtration data were analyzed via a combined model proposed by Ho and Zydney [5]. The plot of J/J_0 versus time, *t,* was fitted to Eq. (1) via a nonlinear optimization using the curve-fitting tool in MATLAB. d^2t/dV^2 versus dt/dV curves of all the filtration data were plotted, and the dominant mechanism was determined according to the following equation.

$$\frac{d^2t}{dV^2} = k\left(\frac{dt}{dV}\right)^n \tag{S1}$$

where t is the filtration time, V is the total filtered volume, and n implies the filtration mechanism, with n=2 for complete pore blocking, n=1.5 for standard blocking, n=1 for intermediate blocking, and n=0 for cake filtration. The required derivatives in Eq. (1) were evaluated in terms of the filtrate flux.

$$\frac{dt}{dV} = \frac{1}{JA} \tag{S2}$$

$$\frac{d^2t}{dV^2} = -\frac{1}{J^3A^2}\frac{dJ}{dt} \tag{S3}$$

where dJ/dt was numerically evaluated by differentiating the flux versus time using the curvefit toolbox in MATLAB® 2014 (Mathworks, Inc.) to obtain the derivative of a series of piecewise cubic polynomials that were fit to the raw data. The exponent n in Eq. (4) was analytically evaluated by differentiating the logarithm of d^2t/dV^2 with respect to the logarithm of dt/dV.

$$n = \frac{d\left[\log\left(\frac{d^2t}{dV^2}\right)\right]}{d\left[\log\left(\frac{dt}{dV}\right)\right]} \tag{S4}$$

The required derivatives were evaluated using Eq. (2) and (3), and the n values were evaluated in different phases throughout the filtration.

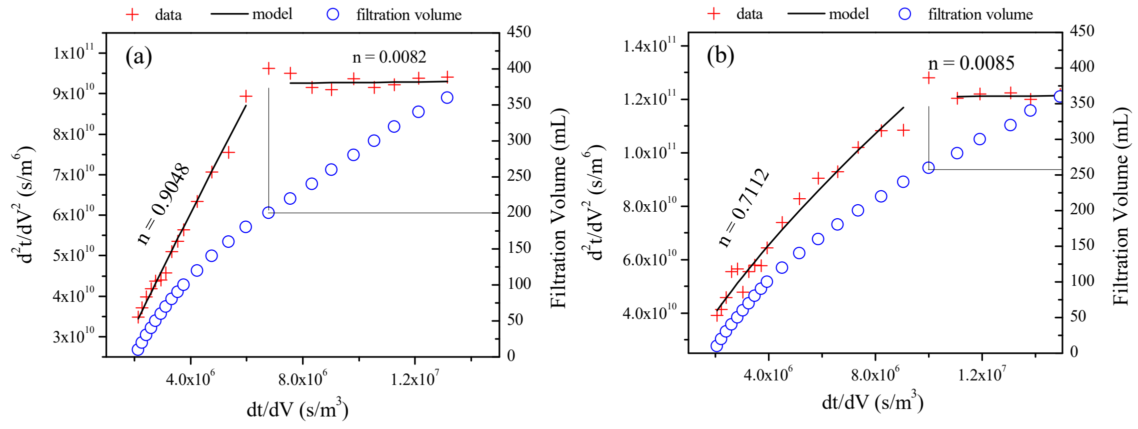

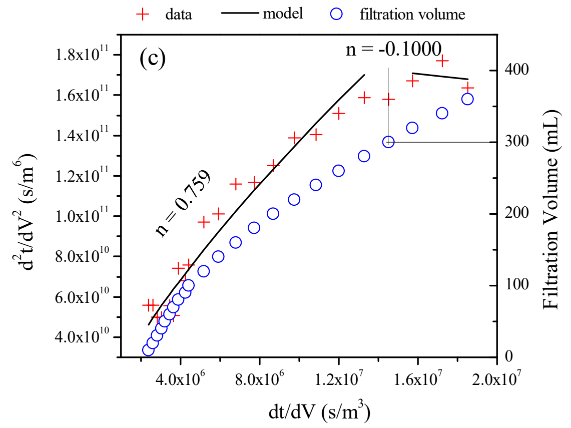

Fig. S4 d^2t/dV^2 vs dt/dV curves for the filtration of reconstituted NOM solutions: (a) NOM, (b) NOM+Na^+, (c) NOM+Ca^{2+}. Values of *n* in eq S4 are evaluatedand shown. Filtration volume versus *dt/dV* curves for the filtrations are also shown.

6. **Characterization of clean and fouled membranes by Attenuated Total Reflection Fourier transform infrared spectroscopy**

Functional groups of foulants on the membrane surface were characterized using an attenuated total reflectance Fourier transform infrared spectrometer (ATR-FTIR, Spectrum One B, Perkin Elmer Inc., USA). The measured range of wavenumber was between 4000 and 500 cm-1.

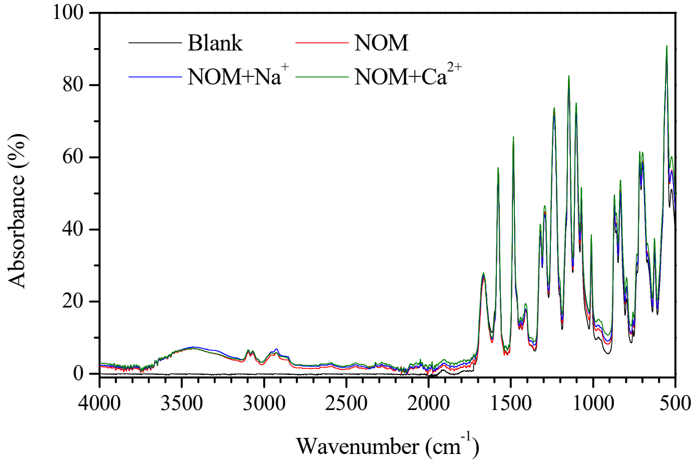

Fig. S5 FTIR spectra for fouled membranes after filtering reconstituted NOM solutions

Reference

[1] C.A. Stedmon, R. Bro, Characterizing dissolved organic matter fluorescence with parallel factor analysis: a tutorial, Limnol. Oceanogr. Methods, 6 (2008) 572-579.

[2] K.R. Murphy, A. Hambly, S. Singh, R.K. Henderson, A. Baker, R. Stuetz, S.J. Khan, Organic Matter Fluorescence in Municipal Water Recycling Schemes: Toward a Unified PARAFAC Model, Environ. Sci. Technol., 45 (2011) 2909-2916.

[3] S.A. Baghoth, S.K. Sharma, G.L. Amy, Tracking natural organic matter (NOM) in a drinking water treatment plant using fluorescence excitation–emission matrices and PARAFAC, Water Res., 45 (2011) 797-809.

[4] N.P. Sanchez, A.T. Skeriotis, C.M. Miller, Assessment of dissolved organic matter fluorescence PARAFAC components before and after coagulation–filtration in a full scale water treatment plant, Water Res., 47 (2013) 1679-1690.

[5] C.C. Ho, A.L. Zydney, A Combined Pore Blockage and Cake Filtration Model for Protein Fouling during Microfiltration, J. Colloid. Interf. Sci., 232 (2000) 389-399.
